# Nursing students’ movement toward becoming a professional caring nurse

**DOI:** 10.1177/09697330241238343

**Published:** 2024-03-16

**Authors:** Turid Anita Jaastad, Venke Ueland, Camilla Koskinen

**Affiliations:** 1040 Åbo Akademi University; 621781 University of Stavanger; 621781 University of Stavanger; 621781 University of Stavanger; 1040 Åbo Akademi University

**Keywords:** Becoming a caring nurse, education, nursing student, systematic review, thematic synthesis

## Abstract

**Background:**

Previous research mainly focuses on how to support nursing students in caring for the patient and on educators’ views of students’ development as professional caring nurses. Against this background, it is important to further investigate nursing students’ perspectives on what it means to become a professional caring nurse.

**Research aim:**

This qualitative systematic review study aims to identify and synthesize nursing students’ perceptions on the meaning of becoming a caring nurse.

**Research design and data sources:**

Systematic data searches were conducted by using the electronic databases MEDLINE (Ovid), CINAHL (EBSCO), Academic Search Premiere (EBSCO), and Philosopher`s Index. In total, 13 studies met the inclusion and quality criteria. The articles were analyzed by a systematic review and a thematic synthesis according to Thomas and Harden.

**Ethical consideration:**

The study followed good ethical practice guidelines outlined in the Northern Nurses’ Federation.

**Findings:**

The analysis resulted in eight descriptive themes and finally in three analytical themes: Becoming is to get in touch with one’s inner ethic or ethos, Becoming is a movement between courage, understanding, and being touched, and Becoming is strengthened through caring role models and a learning culture.

**Conclusions:**

Becoming a professional caring nurse is seen as an ongoing movement toward a deeper understanding of oneself and one’s being and bearing. This movement is enabled when nursing students have a sense of self-awareness, courage to stand in their vulnerability, and reflect on their responsibility, caring attitude, and inner values and ethics. The force of becoming is that the attention is directed beyond self to care for and feel empathy for others in a caring manner. Becoming is released through a caring relationship, external confirmation, and good role models. A lack of external support in the movement can potentially prevent the students from becoming a professional caring nurse.

## Introduction

This study proceeds from the view that caring is a mode of being and a core value of the nursing profession. This starting-point is in line with Martinsen’s^1^ and Eriksson’s^2^ caring theories, where to be a caring nurse is viewed as a mode of being that includes sensitivity to human vulnerability and a will to alleviate the patient’s suffering and ensure the patient’s dignity and absolute value as a human being. According to Matilainen,^3^ and caring science didactics, human dignity refers to a belief in a student’s ability to progress, grow, and become. Several other didactical studies^4–8^ also emphasize that a primary goal of nursing education is to facilitate and support nursing students in caring for the patient, which in turn involves supporting a student’s personal development to become a professional nurse. As caring plays a crucial role in good and ethical nursing care and the well-being of patients,^9,10^ it is seen as essential to further investigate nursing students’ perspectives on their awareness of professional and personal development in becoming caring nurses.

## Background and significance

Previous research shows that nursing education today often places great emphasis on the acquisition of nursing knowledge in the form of clinical/technical skills and competencies, knowledge where the core is to assess the students’ competence according to clear professional standards ensuring that students deliver safe nursing care.^11,12^ Students’ profession-specific knowledge and skills are central to nursing education but the education of caring and nursing students’ awareness of their professional and personal development in the process of becoming a nurse should not be overlooked. Karlsson and Pennbrant^13^ indicate that if nurses are aware of and in touch with their inner core values, it is easier for them to understand and invite a patient into a caring relationship. This is in line with earlier studies^14–16^ that highlight how nursing students who are in touch with their inner core values are more sensitive to the patient’s voice and can invite the patient into a caring relationship. Nursing students’ development and becoming thereby involves professional, personal, relational, and ethical dimensions. According to Scanlon,^17^ becoming is a transformative learning process that affects the entire person and thereby the professional self can be shaped into a professional identity. Also Dall’Alba^18^ supports the idea that becoming professional goes beyond the students’ knowledge and abilities; it encompasses the person they are. There is also a movement in students’ becoming, an idea that becoming is neither something completely “fixed” nor final but a process of becoming professional.^19^ Lundell Rudberg et al.^20^ describe in their study how undergraduate nursing students started their education with dreams and a naive understanding of the profession, and how an understanding of the complexity of the nursing profession gradually developed. Ranjbar et al.^21^ also note how the moral characteristics and professional identity of nursing students develop over a series of phases. When students find and integrate their moral characteristics, these become ingrained both as professionals and as personal selves.

From a teaching perspective, the process of being and becoming a professional caring nurse is emphasized as a crucial aspect of nursing students’ education and training.^22–25^ Dall’Alba and Barnacle^26^ propose that we need an ontological shift in higher education to provide students with the opportunity to reflect on the inter-relationship of knowing-acting-being. We need to enable nursing students to become reflexive and critically aware of themselves in their progress toward becoming professionals. Previous studies^4,27,28^ reveal that directing attention toward becoming a caring nurse can influence the development of nursing students by improving their reflective capacity and self-awareness. According to Ekebergh et al.,^16^ reflective learning and caring are crucial for the development of nursing students’ ability to adopt a caring approach and become both sensitive and sensible nurses.

Previous research mainly focuses on educators’ views on the meaning of student development in the education of professional caring nurses. However, it is important to highlight research on nursing students’ perspectives on the meaning of becoming professional caring nurses.

## Aim

This qualitative systematic review study aims to identify, synthesize, and understand nursing students’ perceptions of the meaning of becoming a professional caring nurse. The research question is as follows: how do nursing students describe their becoming a caring nurse?

## Design and methods

### Design

The study adopts a qualitative research design with an inductive approach to openly deepen the understanding and obtain insights into the unique perspectives and experiences of the students.

### Methodology

The present study is inductive and does not start from a given theoretical perspective but is inspired by Gadamer’s^29^ hermeneutical approach. This study aims to deepen the understanding of the meaning of becoming a professional caring nurse. Also Thomas and Harden’s^30^ systematic review method was selected because it provides an opportunity for interpretation and abstraction.

### Method

A systematic review method, thematic synthesis according to Thomas and Harden,^30^ was used as a method for the systematic data search (collection) and thematic synthesis. The thematic synthesis consists of three stages: (1) coding text; (2) developing descriptive themes; and (3) generating analytical themes.

### Eligibility criteria

The study is implemented in accordance with the Joanne Briggs Institute (JBI) approach to and guidance for synthesizing the evidence of a qualitative research methodology.^31,32^

To guide the search for and extraction of data from qualitative studies, the Participants, Phenomenon of Interest, and Context (PICo) framework was used.^31^ The participants were defined as nursing students, the phenomenon of interest was becoming a caring nurse, and the context was nursing students during their education. Publications were included if the participants were nursing students, if the research adopted a qualitative approach for investigating the issue; nursing students’ understanding of caring and their formation, growth, and becoming. There was no limitation in terms of the year of publication. Publications were excluded if they were not published in English, if they were not peer-reviewed and full-text, if they were literature reviews, if they used quantitative methods or mixed methods, and if the results were not based on nursing students’ perspectives.

### Search strategy

The search strategy was designed in collaboration with the first author and a specialized librarian. Systematic data searches (collection) were conducted in February 2023 and June 2023 in the following electronic databases: MEDLINE (Ovid), CINAHL (EBSCO), Academic search premiere (EBSCO), and Philosopher`s Index. These are the search words that were used in combination or separately: S1. “Nursing students OR student nurse OR undergraduate student nurse”; S2 “becoming” OR “growing” OR “formation” OR “development”; S3 “caring” OR “caring in nursing”; and in combination S1 AND S2 AND S3.

### Search outcome

**Figure 1.**
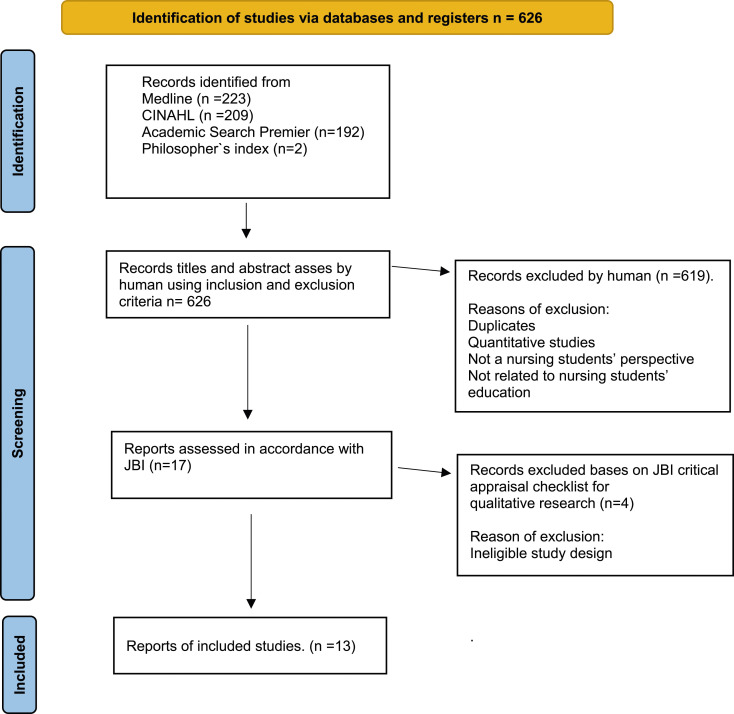
PRISMA 2020 flow chart of the selection of included articles.

The search identified a total of *n* = 626 potentially relevant publications. Titles and abstracts were screened against the inclusion and exclusion criteria by the first author to ensure that all relevant articles were found. The first author systematically reviewed all the articles. The criterion that the publications had to be qualitative studies (not mixed methods) and specifically focus on the student’s perspective resulted in the exclusion of many articles. Numerous articles centered on caring in various contexts, or were based on learning and educators’ perspectives. Based on the abstracts, a total of 619 publications were duplicates or articles that did not meet the inclusion criteria and were duly excluded. Articles were excluded in cases where nursing students’ understanding of their caring, formation, growth, or becoming was not the primary research outcome. After the screening, 17 articles were considered potentially eligible. The search process is presented in the flow chart of the search strategy (Figure 1).

### Quality appraisal

The methodological quality of the 17 included studies was assessed by two authors (T.A.J and C.K). The authors worked independently and followed The Joanna Briggs Critical Appraisal Tools for methodological quality assessment.^32^ After that, the researchers discussed and agreed with their assessments. A total of 13 studies met the criteria for quality. The research methodology was weakly described in four of the studies. Despite this, we decided to include the articles because the research design and methods were logically described and well-conducted. The participants’ perspective was represented adequately across all the included studies. After reading the material we found that the quality was sufficiently large and varied to obtain saturation.^30^ An overview of the studies is provided in Table 1.

**Table 1.** Description of the included qualitative articles.

Author(s)YearCountryJournal/source	Purpose	Data collection participants	Data analysis	Summary of findings
Adamson and Dewar 2015	Describe the use of stories within the curricula to enhance knowledge and skills in compassionate caring.	16 students Action research project whit reflective learning Online discussions.		Stories can initiate this process and help student nurses to understand not only the needs of others, but their own expectations and values, which in turn can inform how they plan and deliver person centered compassionate care.
Albinsson et al. 2020	The aim of the study is to explore how nursing students talks about their notions on interactions in the relationship between nurse and patient. Applying thematic, and using.	22 students in their first semester. Individual interviews.	Qualitative content analyses.	The results provide new insights. Into the interactions in the nurse-patient relationship, seen from the perspective of beginner students.
Callister et al. 2009 USA Nursing ethics	Describe ethical reasoning in baccalaureate nursing students enrolled in a nursing ethics course.	Reflective clinical journals. 70 nursing students.	Qualitative content analysis.	The development of critical thinking and ethical reasoning within the framework of knowing and connecting is essential in nursing education.
Crigger 2001 USA Journal of Advanced Nursing	Understand the process, particularly. Engrossment, and to facilitate students’ development of caring relationships.	Nonstructured interview. 13 nursing students.	Data analysis consisted of six steps.	The findings suggest that caring is a process comprised of mental choices and affective responsiveness.
Fitzgerald and Clukey 2021 USA Journal of Nursing Education	To examines the meaning of nursing professional identity development from the perspectives of both associate degree nursing students and baccalaureate degree nursing students in their final semester.	Individual interviews Focus groups interviews 21.	Method of analysis of phenomenological data.	The participants described the nurse with a strong professional identity was the emphasis on personal characteristics or ingrained traits and not on actions or skills. The students described someone who cares about the patient, who has integrity, and who is able to work as part of a team.
Holland 1999 England Journal of Advanced Nursing	Explored the nature of the transition experienced by student nurses in their journey to becoming qualified nurses.	Open-ended questionnaires interviews.	Thematic analysis	The findings revealed two ‘in limbo’ states, a ritualized transition phase, and that the rationale for ‘learning to become a nurse’ retains the idealized and vocational imagery of nursing as helping and caring for sick people.
Jaastad et al. 2022 Norway Scandinavian Journal of Caring Sciences	Gain knowledge of the meaning of reflection in first-term nursing education, and how reflection grounded in caring theory can deepen the students’ understanding of caring and their professional formation of becoming a caring nurse.	Written reflective descriptions. 64 nursing students.	Thematic analysis	Appropriation of caring theory provides a foundation for the nurse students to see themselves within a broader perspective and is important for mutual support in the professional formation of becoming a caring nurse.
Lindberg et al. 2018 Sweden Nurse Education Today	To describe the experiences of learning about caring science by participating in reflective seminars that were integrated into courses during a 3-year nursing education program.	Group interviews 23 nursing students.	The analysis process was informed by the methodological principles of reflective lifeworld research.	The study contributes to the understanding of reflective seminars grounded in lifeworld theory as a didactic strategy that enables students to increase their knowledge of caring science and develop their reflective skills.
Pedersen and Sivonen, 2012 Denmark – Finland Nursing Ethics	Get a deeper understanding of student nurses’ experiences of personal caring ethics.	Interview 24 student nurses	Phenomenological–hermeneutical approach	The students’ ethical caring ideals served as fixed reference points in their ethical development, but their ideals were at risk of decline. The students reflected on the barriers for performing ethical care and nurtured their ethical ideals by providing ethical care in secret.
Teskereci, et al. 2019 Turkey Nurse Education in Practice	To explore the initial experiences of first-year nursing students during clinical practices.	Written diaries. 12 nursing students.	Thematic analysis	It was found that students needed support from the faculty and nurses to develop a caring vision and professional identity.
Mackintosh, 2006 UK. International Journal of Nursing Studies	To identify the effect time has on participants’ attitudes and views of care and becoming a nurse, during pre-registration nurse training.	Semi structured interviews random sample of 16 pre-registration student nurses.	Qualitative analysis	The study identifies an under recognized dichotomy between the caring ethos of professional nursing and the professional socialization processes student nurses are subject to, which directly mitigate against the individual nurses abilities to care.
Sandvik et al. 2014 Finland Nurse Education in Practice	Deepen the understanding of student learning and development in becoming a nurse.	Focus group interviews		Caring relationships can be seen as the foundation for student learning and development and as a prerequisite for becoming a nurse. Learning is not merely a skills-acquisition procedure but a transforming experience for students, who feel that their whole existence has been altered.
Sandvik et al. 2015 Finland	To deepen the understanding of student nurses’ processes of understanding and becoming nurses.	Focus group interviews. 21 nursing students.	Phenomenological hermeneutical method.	Understanding and becoming are ongoing processes of appropriation, thus altering students both professionally and personally. Understanding and becoming can be perceived as the hearth of the matter in nurse education.

### Synthesis and analysis

The thematic synthesis following Thomas and Harden^30^ included three stages: line‐by‐line coding of the text, development of descriptive themes, and generation of analytical themes. While the development of descriptive themes remains “close” to the primary studies, the analytical themes represent a stage of interpretation whereby the reviewers generate new interpretive constructs or explanations. The preliminary synthesis was conducted by the first author. In the first stage, the author used line-by-line coding and organized the key components. The second stage involved organizing and abstracting the key components into descriptive themes. Through a process of reflection and discussion, and based on the aim, the authors formulated eight descriptive themes that unified the included studies. In the final stage, the first author used descriptive themes in the interpretation of a new thematic synthesis that went beyond the original studies. Three analytic themes emerged in the analyzing process, which constituted a higher level of abstraction. In the generation of analytical themes, mainly caring theories were applied to add a deeper understanding to the abstraction and interpretation. Finally, all three authors (TAJ, VU, and CK) discussed the descriptive and analytical themes until a consensus was reached.

### Ethical considerations

This study, conducted in 2023, followed good ethical practice guidelines outlined in the Northern Nurses’ Federation.^33^

## Findings

### Descriptive themes

Eight descriptive themes emerged in the analysis: *Awareness of one’s inner caring values, The courage to be the person and nurse one wants to be, Awareness of one’s own responsibility, To be touched by and have empathy for the other, To confirm and understand human vulnerability, To understand the meaning of nursing and caring, Becoming through a caring relationship, Becoming through appropriation of knowledge, skills, values, and a caring attitude*.

*Awareness of one’s inner caring values* highlights the ongoing process of developing a deeper understanding of one’s personal values, beliefs, and being. Studies showed that when students are given opportunities to dive into themselves, to listen, hear and get to know themselves, they develop a deeper self-awareness and understanding of how to recreate their practice, to re-image themselves.^34–43^ For nursing students, awareness of one’s inner caring values is strongly connected to their incorporation of caring as a value base, which then becomes visible in their bearing and actions. Nursing students explicitly reported that their awareness of their ethical inner caring values guided them in their encounter with patients and strengthened them in their efforts to be a caring nurse who treat the patients as unique and whole persons. For example, the student’s reflection grounded in caring science theory reinforces the student’s self-understanding and ethical awareness. Through this process, students cultivate an enhanced sense of self-awareness, enabling them to evaluate whether their being and values align with their desired goals and values.^38,39^

*The courage to be the person and nurse one wants to be* focuses on nursing students’ courage of being in accordance with their inner caring values.^34–36,38,40,41,43–45^ Nursing students highlight how reflection gives them the courage and strength to stand up for their moral beliefs when meeting with ethical issues. The students articulate the necessity for courage to truly witness the patients’ suffering, and how this courage empowers them to provide ethical nursing and caring.^41,45^ Furthermore a strong will and courage to provide care are crucial prerequisites for the student’s professional development.^35,40^ Moreover, the students’ understanding fosters confidence and supports their courage to trust themselves. These students emphasize that their personal growth evolves from their confidence and understanding.^36,43^

*Awareness of one’s own responsibility* emphasizes the student’s self-awareness and understanding of being a responsible caring person and nurse.^35–38,40,41,43–45^ Nursing students relate that they have a responsibility for their own personal and professional development and that responsibility entails continuously demonstrating a willingness for reflection and evaluation to improve one’s own doing and being. This understanding, in turn, fosters an internal motivation to mold oneself to be a responsible caring nurse and human being. The students further elaborated this by conveying that taking responsibility and engaging in personal development strengthens their abilities to take responsibility for alleviating patient suffering.^41,43^ Taking responsibility for alleviating patient suffering encourages students to reflect on their doing and being. Such encouragement, in turn, promotes personal and professional growth and enhances students’ ability to understand themselves and improve their nursing practice.

*To be touched by and have empathy for the other* underscores the importance of being open to being moved in a way that contributes to creating a meaningful and empathetic nursing practice in encounters with patients.^34,35,37,40,41,44–46^ The students describe how they have been emotionally affected by the patient’s pain, suffering, and challenges. When students are touched by the patient, willingness arises in them to understand the patient’s feelings and challenges. The studies^35,41,44^ underline that the students’ understanding of the patient’s situation help them to learn to express themselves clearly, use empathetic communication, and actively listen to the patient’s concerns. By being sensitive to the patient’s situation, the nursing students better understood how they could facilitate the alleviation of patient suffering.

*To confirm and understand human vulnerability* refers to when nursing students develop a deeper awareness and understanding of their own and other’s vulnerability.^34,35,38–41,43–45^ The students’ vulnerability was activated in the meeting with the patient’s suffering and became a source of development with focus on the patient.^41^ Students utilize their own vulnerability and imagination in developing a deeper understanding of the patient as vulnerable and unique.^38,39^ This fosters a deeper understanding of patients’ needs and feelings and strengthens their understanding of how to support and comfort the patient. The included studies also showed that students are constantly confronted with their own vulnerability and uncertainty while striving to be a caring nurse when meeting the patients.^34,35,40,45^

*To understand the meaning of nursing and caring* stresses that becoming is strengthened when students gain a deeper understanding of how their nursing and caring can alleviate patient suffering, promote healing, and health.^34–38,40–44^ The students in the studies, by reflecting on their own nursing practices, express that they developed a deeper understanding of what nursing and caring truly mean for other people. They became more aware of the values and knowledge that are important to them and how they want to be as nurses. This process of reflection gives them the opportunity to learn from their experiences and develop a deeper knowledge and insight into what nursing and caring truly mean. The nursing students experience an inner motivation and find meaning when they realize that their caring and nursing make a positive difference in patients’ lives.^41,42^ By reflecting on the deeper dimensions of nursing and caring, they become more confident in their role as nurses and grow into the nursing profession.

*Becoming through a caring relationship* spotlight how, for nursing students, role models of caring play a crucial role in becoming a caring nurse. Studies^34–39,41–43,45,46^ emphasize that when nursing students have role models of caring in practice, they can recognize the importance of these values and use them as guidance for their own nursing practices. The students express that by observing the ways in which others care for patients in a caring or non-caring manner has given them a deeper understanding and self-awareness of how they want to be and present themselves in their interactions with patients.^36,41^ This personal insight can strengthen their ability to provide care and create meaningful caring relationships with patients in future practice. The students indicate that caring relationships with fellow students and teachers can contribute to shaping their attitudes, values, and skills, preparing them to be caring and supportive nurses in their future profession.^38,39^ In the studies, caring refers to the importance of nursing students being treated with trust and confidence in their ability to develop and learn to become nurses.^42,45^

*Becoming through appropriation of knowledge, skills, values, and a caring attitude* refers to how nursing students are formed into caring nurses through an integration of knowledge, skills, values, and attitudes that promote patient well-being and healing.^34,38,39,41–44^ The students emphasize that nursing combines an emotional, caring aspect with a scientific, analytical aspect. They recognize that nursing students need to balance these two aspects to provide optimal care and achieve positive outcomes for patients.^34,36,39^ The students relate how reflection supports their understanding of caring theory and caring in nursing, which is crucial for becoming nurses.^38,39^ They explain that the appropriation of caring science theory enhances their ethical awareness. They become more conscious of the values and knowledge that are essential to them as nurses and how to combine caring with professional competence and knowledge.^41,43^

### Analytical themes

The findings generated three analytical themes: *Becoming is to get in touch with one’s inner ethic or ethos*. *Becoming is a movement between courage, understanding and being touched* and *Becoming is strengthened through caring role models and a learning culture.*

#### Becoming is to get in touch with one’s inner ethic or ethos

The findings of this study show that the students’ becoming is an inner movement where they explore or come into contact with their inner ethic or ethos. According to Eriksson,^47^ ethics and ethos represent a bearing, and a stance, and are regarded as the nurse’s innermost core and an important foundation for becoming and formation. In this inner movement, the students begin to recognize, examine, and understand what is important to them in terms of caring and their own personal values as professional nurses. By reflecting on their own being and inner values, and how these affect relationships and interaction with others, students become more aware of themselves as caring nurses. This self-understanding sets in motion an inner movement in which they develop the will and courage to make decisions and act according to their inner ethic or ethos. These findings resonate with those of Hilli and Eriksson,^48^ which show that human beings who are in contact with their ethos, the self, feel at home, and have the courage to follow their heart’s inner voice. According to the students, being in touch with their inner ethics or ethos represents a significant driving force and inspiration that encourages them to be honest, open, and authentic in their interactions with themselves and others. This ethical self-understanding thus plays a significant role in their development as caring nurses.

#### Becoming is a movement between courage, understanding and being touched

Another interesting finding is that courage plays a pivotal role in shaping the students’ formation; it involves the integration of their professional qualifications with their personal qualities. When students overcome fear, anxiety, and discomfort and delve deep into their own values and beliefs, their courage begins to grow. These findings agree with Gibson^49^ and Numminen,^50^ underscoring how courage constitutes a fundamental aspect of nurse students’ ethical proficiency and a trait that can be cultivated and nurtured. The findings show that by being courageous, the students recognize their own vulnerability and develop a more profound understanding of being a whole person. This understanding sets in motion an inner movement where they become more aware of and sensitive to other people’s vulnerability and suffering. This is in line with Martinsen,^51^ who notes that nurses’ own vulnerability, suffering, and sore points shape their courage to be witnesses to the patient’s suffering and to show willingness to care for the other. The results reveal that being a witness to other people’s pain and suffering can be challenging for students and requires that they stand in the uncomfortable and unknown, and that they dare to show commitment to face the other’s vulnerability. These findings resonate with the results of the studies by Thorup et al.^52^ and Lachman^53^ showing that courage to act is a required inner quality and a first step toward an existential caring encounter. On this existential level, in facing the unpredictable, courage contributes to the nurturance of personal and professional development.

#### Becoming is strengthened through caring role models and a learning culture

The findings reveal that nursing students need support in their formation toward becoming caring nurses. Observing, and learning from caring role models has a profound impact on their own personal and professional development. Educators are role models for students because their thought, language, bearing, and actions reflect their inner perception and understanding of reality and view of science and caring in nursing.^6^ These role models help the students better comprehend the process of forming their own identity as future nurses and understanding what it means to being a caring nurse.^54^

According to the students, theories facilitate their understanding of what they are doing, why and what the consequences are, and how to evaluate their being and action. Learning cultures where students have the opportunity to reflect and intertwine theory and practice are required and promote students’ appropriation. These findings agree with Ekebergh^55^ and Hörberg et al.^56^ who suggest that when students explore and understand their core values and sense of self, they can more easily develop a deeper understanding of nursing and caring. The process of reflection, starting with self-image, allows students to express their personal experiences in relation to theory and caring. A process of self-awareness that involves both the inner and external aspects of the student’s life can lead to increased self-confidence, understanding, and maturity.

## Discussion

The fundamental driving force in becoming is willingness and courage to be affected and to connect with self and others. In this deeper inner movement, the students explore their inner world and reflect on their being, and they develop a more profound sense of self-awareness and understanding of caring and of themselves as caring nurses. This is in line with Dall’Alba,^18^ who concluded that the self-transformation into becoming a professional is not solely an individual or isolated endeavor. In their daily lives, nursing students are intertwined with others, often interpreting themselves through the perceptions and reflections of those around them. Supporting our findings is the recognition that reflection sets the students’ internal development in motion. Through this self-reflection, students attain a heightened awareness of their ethical stance, and of how their actions, attitudes, and being influence the way patients experience their caring and nursing. A comparison can be made with Alvsvåg and Martinsen,^57^ who emphasize that an essential aspect of being a caring nurse is to maintain an open-minded approach to various forms of knowledge and to have a conscious and reflexive approach to oneself as a caring nurse. This perspective is shared by Dall’Alba^18^ in her description of the developmental process of becoming a professional, which encompasses both the acquisition of knowledge and being.

By reflecting on their thoughts, doings, and experiences in relation to the patient, nursing students not only developed their skills, but also attained a deeper understanding of themselves as professional caring nurses. This can be compared to the process of becoming a professional as described by Scanlon,^17^ who characterizes it as an evolutionary, iterative process in which individuals develop their professional self, a professional identity. Professional identities are shaped and sustained through continuous self-negotiation, a process where individuals are defined by others and, in turn, define and redefine themselves over time.

Our study indicates that the student’s progression toward becoming a professional caring nurse is strengthened and motivated through the development of self-awareness. By being self-aware, students undertake the crucial responsibility of shaping themselves professionally, ethically, and existentially. This aligns with the results of Li et al.,^58^ who concluded that guiding and nurturing students’ self-awareness is crucial as it can support them in developing a more profound understanding and wisdom of themselves as caring nurses.

A thriving educational culture with a supportive and inclusive learning environment can facilitate the inner and external aspects of becoming a professional caring nurse. For Hilli et al.,^59^ a caring relationship founded on caring ethics is regarded as the cornerstone in assisting students in their development to become a nurse. This approach encourages reflection and critical thinking, thereby facilitating the students’ dual development of both professional and personal aspects. It aligns with the perspective of Dall’Alba and Barnacle,^26^ who emphasize that an ontological shift in higher education enables students to contemplate the interconnectedness of knowing, acting, and being. The findings indicate the importance of maintaining awareness of nursing students’ vulnerability during the movement toward becoming a professional caring nurse. This movement represents a profound self-understanding, and at the same time, the nursing students must also redirect their attention beyond themselves to care for others in a caring manner. Heggestad et al.^60^ describe how vulnerability is a crucial aspect that nursing education needs to acknowledge and consider, to create a conducive learning environment that facilitates nursing students’ professional and personal professional development. The students also stress the value of role models for their becoming. The role models’ personal and professional being and bearing, not just their knowledge, affect and model for students who they will become as caring nurses. According to Salminen et al.,^61^ educators are role models for students’ ethical bearing because their perception and understanding of reality and their worldview and scientific vision are always reflected in their thoughts, language, bearing, and actions.

## Conclusion

In conclusion, the meaning of becoming a professional caring nurse is seen as an ongoing movement toward a deeper understanding of oneself and one’s being and bearing. This movement is enabled when nursing students have a sense of self-awareness, courage to stand in their vulnerability, and to reflect on their responsibility, caring attitude, and inner values and ethics. The force of becoming is that attention is directed beyond self to care for and feel empathy for others in a caring manner. Becoming is released through a caring relationship, external confirmation, and good role models. A lack of external support in the movement can potentially prevent the students from becoming a professional caring nurse.

### Implications for nursing education and research

The findings highlight the importance of a caring educational culture consisting of a supportive and inclusive learning environment that facilitates the inner and external aspects of becoming a professional caring nurse. The experience of caring and support within the educational environment can contribute to students’ professional and personal growth.

Based on our findings, we recommend that the nursing curriculum progressively supports the students’ movement toward becoming a caring nurse. Future research should explore how the employment of diverse educational methods and didactic approaches can support nursing students in shaping their personal and professional identity to become a professional caring nurse.

### Strengths and limitations

The strength of this study lies in the fact that several researchers participated in the various steps and the articles were reviewed based on given criteria. Although the study was carried out based on a given methodology, method, and search strategy, there is always the possibility in literature reviews of unintentionally leaving out certain aspects of the studies that are included.^30^ It is also crucial to recognize that this review reflects the authors’ interpretation of the studies. Different outcomes may arise from researchers with varying interests in the field.
